# A Rare Case of Dengue Fever Presenting With Acute Disseminated Encephalomyelitis

**DOI:** 10.7759/cureus.10042

**Published:** 2020-08-26

**Authors:** Umar Farooque, Bharat Pillai, Sundas Karimi, Asfand Yar Cheema, Noman Saleem

**Affiliations:** 1 Neurology, Dow University of Health Sciences, Karachi, PAK; 2 Neurology, Amrita Institute of Medical Sciences, Kochi, IND; 3 General Surgery, Combined Military Hospital, Karachi, PAK; 4 Medicine, Lahore Medical and Dental College, Lahore, PAK; 5 Forensic Medicine, Sahiwal Medical College, Sahiwal, PAK

**Keywords:** acute disseminated encephalomyelitis, atypical manifestations, dengue fever, neurological complication, dengue fever/complications, humans

## Abstract

Dengue fever is a viral infection transmitted by mosquitoes with a clinical spectrum that ranges from asymptomatic infection to dengue shock syndrome. Neurologic manifestations are rare. We report a case of dengue fever presented with acute disseminated encephalomyelitis. An 18-year-old boy presented with high-grade fever, generalized headache for three days, intermittent altered sensorium, nausea, and vomiting for one day. Dengue-IgG and Dengue-IgM were positive. Magnetic resonance imaging (MRI) showed abnormal signal intensity areas in the bilateral deep white matter at centrum semiovale more on the right side, which seemed hypointense on T1 and hyperintense on T2 and fluid-attenuated inversion recovery (FLAIR) images, with open ring enhancement on contrast-enhanced T1 image, and peripheral diffusion restriction on diffusion-weighted 1 (DW1) image. These features were suggestive of acute disseminated encephalomyelitis. He improved within a week of taking IV methylprednisolone 1 g once daily for five days and supportive care. Follow up MRI after three weeks showed the resolution of all abnormalities. Thus we conclude that patients with acute disseminated encephalomyelitis should be checked for dengue fever, especially in areas of high prevalence, for early diagnosis and appropriate treatment and to prevent excessively aggressive surgery and/or treatment for such abnormal MRI findings.

## Introduction

Dengue fever is a common mosquito-borne viral infection in the tropics. It is caused by a flavivirus. Its clinical continuum ranges from as mild as asymptomatic infection to as severe as dengue shock syndrome. Neurologic manifestations are very unusual and occur in 2.6% to 5% of patients [1,2]. These include encephalopathy, encephalitis, Guillain-Barre syndrome (GBS), myelitis, meningitis, acute disseminated encephalomyelitis, facial and ulnar mononeuropathy, acute hypokalemic quadriparesis and stroke, both ischemic and hemorrhagic [2-8]. Acute disseminated encephalomyelitis following dengue fever is extremely rare and very few cases have been documented. We report one such case from Karachi, Pakistan.

## Case presentation

An 18-year-old boy came in the outpatient department with high-grade fever, generalized headache for three days, recurrently altered sensorium, nausea, and vomiting for one day. There was no other significant history.

Clinical examination on admission revealed a temperature of 101^o^F, a pulse of 132/min, and blood pressure of 90/40 mmHg. The neurological examination did not reveal any significant abnormality. The rest of the systemic examination was also within normal limits.

Investigations revealed hemoglobin 13.9 g/dL (normal range - 13.2-16.6 g/dL), total leukocyte count 4800 cells/mcL (normal range - 3,400 to 9,600 cells/mcL) with neutrophils 70% (normal range - 55-70%), lymphocytes 21% (normal range - 20-40%), eosinophils 2% (normal range - 1%-4%), and monocytes 7% (normal range - 2-8%), and platelets 124,000/mcL (normal range - 135,000 to 317,000/mcL). Liver enzymes were aspartate aminotransferase (AST) 30 U/L (normal range - 8-48 U/L), alanine aminotransferase (ALT) 36 U/L (normal range - 7-55 U/L), and alkaline phosphatase (ALP) 280 U/L (normal range - 40-129 U/L). Serum albumin concentration, prothrombin time (PT), activated partial thromboplastin time (APTT), and international normalized ratio (INR) were normal. HBsAg, anti-HCV IgG, anti-HAV IgM, and anti-HEV IgG were non-reactive. Random blood sugar, urea, creatinine, and electrolytes were normal. The urine routine examination was normal. The malaria test was negative. Dengue-IgG and Dengue-IgM were positive. Cerebrospinal fluid (CSF) analysis showed a cell count of 36 cells/microliter with polymorphs 20% and lymphocytes 80%. CSF proteins were 125 mg/dL and glucose was 78 mg/dL. CSF gram staining and culture were negative. CSF polymerase chain reaction (PCR) was negative for herpes simplex 1 & 2. Ribonucleic acid (RNA) of dengue virus or virus isolation by the PCR method was not possible due to a lack of investigation facility.

**Figure 1:**
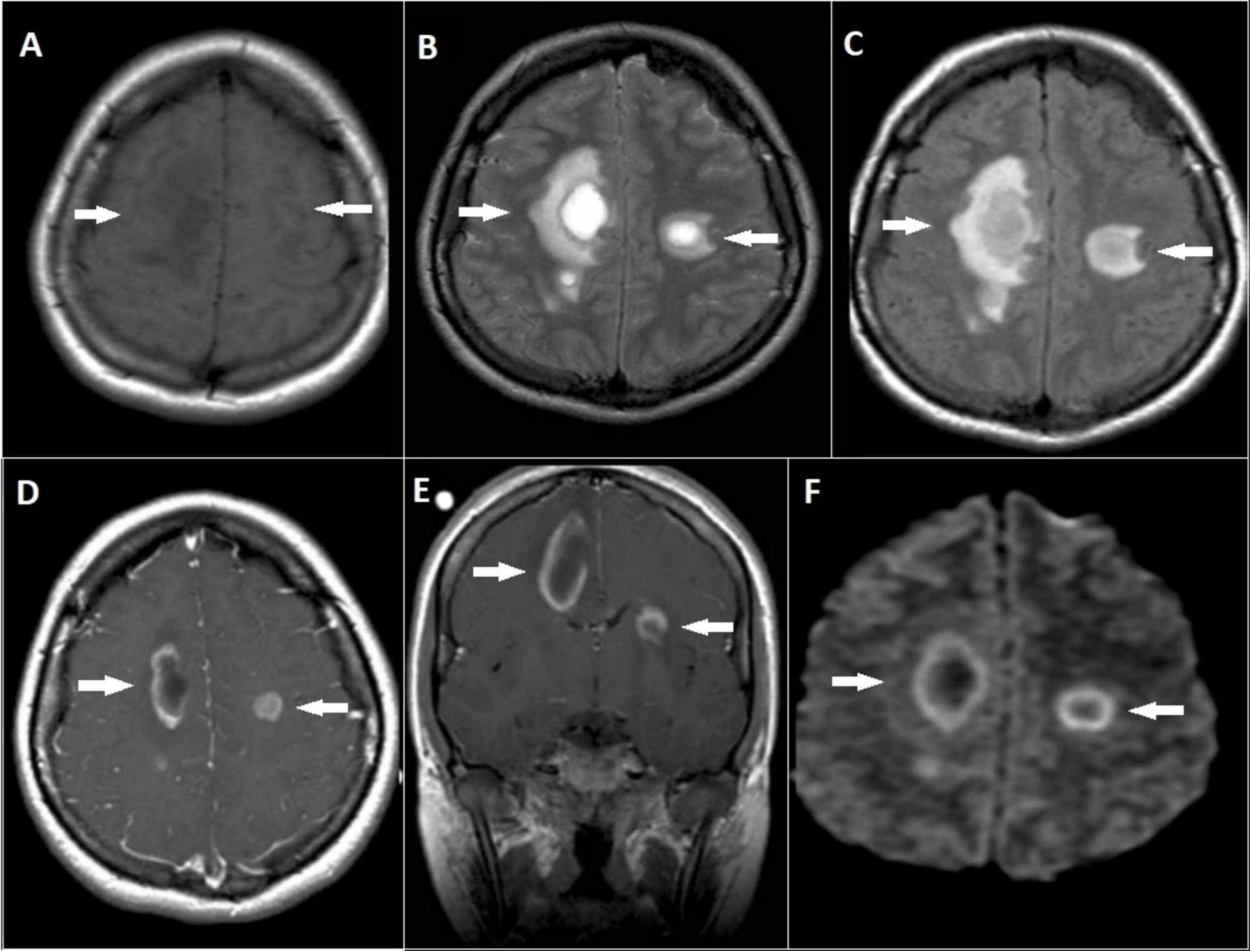
Magnetic resonance imaging of the head A - T1 axial scan showing hypointense areas in bilateral deep white matter at centrum semiovale and more marked on right side. B & C - Fluid-attenuated inversion recovery (FLAIR) and T2 axial scans showing hyperintense areas in bilateral deep white matter at centrum semiovale with perilesional edema and more marked on right side. D & E - Contrast-enhanced T1 axial and coronal scans showing open ring enhancement of the lesions. F - Diffusion-weighted 1 (DW1) axial scan showing peripheral diffusion restriction and no diffusion restriction in the center of the lesions.

Magnetic resonance imaging (MRI) showed bilateral asymmetric abnormal signal intensity lesions in the deep white matter at centrum semiovale with perilesional edema more on the right side, which appeared hypointense on T1 and hyperintense on T2 and fluid-attenuated inversion recovery (FLAIR) scans. The post-contrast T1 scan showed open ring enhancement and the diffusion-weighted 1 (DW1) scan showed peripheral diffusion restriction with no central diffusion restriction. These features indicated acute disseminated encephalomyelitis and are shown in Figure 1.

He was admitted to the hospital and was given intravenous methylprednisolone 1 g for acute disseminated encephalomyelitis once daily for five days, oral acetaminophen 1000 g for fever and headache twice daily for seven days, intravenous normal saline and oral fluids to keep him hydrated and was advised bed rest. He was discharged from the hospital after one week with advice to take rest, drink plenty of water, and take oral acetaminophen when needed. Follow up was after three weeks. MRI was repeated at follow up that showed significant improvement of all previous abnormalities.

## Discussion

The purpose of this case report is to draw attention towards dengue fever being a rare but possible cause of acute disseminated encephalomyelitis.

Dengue virus infection is one of the world's emerging infectious diseases. It was a major viral illness transmitted by mosquitoes and affected humans in 2005 [9]. Each year around 50-100 million cases of dengue fever and 500,000 cases of dengue hemorrhagic fever (DHF) are reported that cause about 24,000 deaths [10].

Dengue fever is now endemic in Pakistan. Its first major outbreak was reported in 1994-1995. During 2005-2006, a second epidemic occurred in the country with 3,640 patients of dengue fever admitted in different hospitals and 40 deaths, making it the biggest outbreak of that time. Since then, it has occurred every year and the city of Karachi is the most affected area. In 2011, Pakistan’s eastern province of Punjab was hit by another epidemic of dengue fever that killed around 365 people and 21,597 cases of dengue fever were reported and it was the world’s biggest epidemic of dengue fever ever [11,12].

Neurologic complications are uncommon in dengue fever. These neurological manifestations of dengue fever can be due to neurotrophic effects of the dengue virus and the systemic effects of the dengue infection or can be immune-mediated. Acute disseminated encephalomyelitis is an immune-mediated disorder of the central nervous system (CNS) that starts after a viral infection or immunization with a rapid onset of neurologic symptoms and signs within days to weeks. Histopathological examination of the central nervous system in acute disseminated encephalomyelitis shows the involvement of the white matter, with the infiltration of monocytes, neutrophils and lymphocytes, and perivenous demyelination [11-14].

## Conclusions

Acute disseminated encephalomyelitis is an unusual but possible presentation of dengue fever. Therefore, any patient with neurologic signs and symptoms, and high-grade fever, especially in an endemic area of dengue fever, should be checked for dengue virus serology and treated accordingly. This approach can help prevent not only unreasonably aggressive surgery or treatment for abnormal MRI findings but also the late diagnosis of dengue fever and its complications, which can improve morbidity and mortality.
